# In vivo trial of bioresorbable mesh cages contained bone graft granules in rabbit femoral bone defects

**DOI:** 10.1038/s41598-024-63067-y

**Published:** 2024-05-30

**Authors:** Toshiki Yanagisawa, Koichiro Hayashi, Akira Tsuchiya, Ryo Kishida, Kunio Ishikawa

**Affiliations:** Department of Biomaterials, Faculty of Dental Science, Kyushu University, 3-1-1 Maidashi, Higashi-ku, Fukuoka, 812-8582 Japan

**Keywords:** Implants, Biomedical materials

## Abstract

Bone graft granules implanted in bone defects come into physical contact with the host bone and form interconnected porous structure. However, there exists an accidental displacement of granules to unintended locations and leakage of granules from bone defects. Although covering the defect with a barrier membrane prevents granule emanation, this procedure is troublesome. To resolve these problems, we fabricated bioresorbable mesh cages (BRMc) in this study. Bone graft granules composed of carbonate apatite alone (Gr) and bioresorbable mesh cages (BRMc/Gr) introduced the bone graft granules and were implanted into the bone defect in the rabbit femur. Micro-computed tomography and histological analysis were conducted at 4 and 12 weeks after implantation. Osteoprogenitors in the bloodstream from the host bone passed through the pores of BRMc, penetrated the porous structure of graft granules, and might interact with individual granules. Then bone remodeling could progress actively and new bone was formed. The new bone formation was similar to the host bone at 12 weeks and there were minimal signs of local tissue inflammation. BRMc/Gr could reduce the risk of unwanted new bone formation occurring due to loss of granules from the bone defects compared with Gr because BRMc enclosed granules and prevent granules leakage from bone defects and BRMc could not induce unfavorable effects to forme new bone. Additionally, BRMc/Gr could keep granules assembled in one place, avoid displacement of granules to unintended locations, and carry easily. These results demonstrated that BRMc/Gr was effective in bone regeneration and improved clinical handling.

## Introduction

The treatment for various bone diseases, such as cysts and benign and malignant tumors, can often result in the development of bone defects^1–5^. When bone graft granules such as autologous, hydroxyapatite [Hap: Ca_10_ (PO_4_)_6_ (OH)_2_] and β-tricalcium phosphate [β-TCP: Ca_3_ (PO_4_)_2_]^6–8^ are implanted in bone defects, these graft granules form an interconnected porous structure which may facilitate cellular and vascular invasion^9,10^. Technical challenges with the present delivery systems include the accidental displacement of the granules to unintended locations, leakage, and loss of granules. The loss of granules hampers bone regeneration^11^. In contrast, additional bone augmentation is one of the standard treatments for dental implantation when the bone volume is insufficient^12–14^. Traditionally, additional bone augmentation is accomplished with barrier membranes to inhibit soft tissue ingrowth and create and maintain a closed environment where osteogenesis may take place relatively unimpeded. The alveolar ridge space can be preserved by adding granules that act as a scaffold to promote new bone formation^15–17^. The membrane also prevents granule emanation. However, if the membrane were no pore, it could prevent entry of cells related to osteogenesis migration into graft and affect bone regeneration. In an earlier study, a titanium mesh was used to keep the form of implanted granules and permitted the passage of osteogenetic cells from the surrounding tissues because of the porous structure^18^. However, as it was not made of biodegradable material, it required a second surgery for its removal. In another study, a bioresorbable soft mesh provided a temporary supporting surface. It was hypothesized that a soft mesh would also be useful for retaining granules^19,20^. Similarly, natural coral skeleton (NCS) granules covered by polyglactin mesh were implanted in critical-sized rat craniotomy defects^11^. It was concluded that bone volume from granules covered by the mesh was more significant than that of granules not covered by mesh because the granules covered by mesh were retained at the defects site. In another study, continuity defects in the canine mandible were reconstructed using a poly (l-lactide) mesh tray and granules cancellous bone and marrow (PCBM). The mesh served as a framework for the implants and permitted the passage of nutrients and blood vessels from the surrounding tissues into the graft to promote new bone formation^21^. However, despite the mesh being fixed with lateral sutures or stainless-steel wire, granules may sometimes leak from bone defects. Although numerous variations of surgical procedures exist, devised to achieve better results, most of these are technically more complicated and must be solved effectively.

We fabricated bioresorbable mesh cages containing bone graft granules (BRMc/Gr) in this study. The mesh cages were used Vicryl mesh knitted type (Ethicon, Somerville, NJ, USA).

Vicryl mesh is fully biodegraded into lactic and glycolic acid by hydrolysis and absorbed after 60 to 90 days in vivo^22^. We hypothesized that if the granules were contained within the mesh cages, the emanation of granules from the bone defect could be prevented, and clinical handling could be improved due to easy carry. We hence introduced carbonate apatite {CO_3_Ap; Ca_10_ − x[(PO_4_)_6_ − x(CO_3_) x] (OH)_2_ − x} granules as the graft granules. Carbonate apatite was approved as an artificial bone substitute which can be used in all dental and maxillofacial fields, including those adjacent to dental implants. In Japan, these are available as Cytrans Granules® (GC Corporation, Tokyo, Japan). Carbonate apatite has good osteoconductivity and a quick bone replacement^10,23–30^. However, whether osteoprogenitor cells pass through the mesh cages and form new bone is unknown if there were mesh cages between the host bone and graft materials (granules). Therefore, we investigated the radiographic and histological features of granules alone (Gr) and BRMc/Gr when the sample was grafted into bone defects in rabbit femur. Furthermore, we evaluated bone formation and leakage of granules from bone defects and compared Gr with BRMc/Gr.

## Materials and methods

### Preparation of CO_3_Ap granules

Ca (OH)_2_ powder (Wako Chemical Co. Ltd., Osaka, Japan) was placed in a stainless-steel mold and pressed uniaxially under a pressure of 5 MPa from an oil pressure press machine (Riken Power; Riken Seiki, Tokyo, Japan). The Ca (OH)_2_ compact discs (10 mm in diameter and 6 mm in thickness) were treated in a stream of CO_2_ gas saturated with water vapor at room temperature for 2 weeks for carbonation. The obtained CaCO_3_ blocks were crushed with a hammer and sieved to collect particles between 300 and 600 μm in size. The granules were immersed in 1 mol/L Na_2_HPO_4_ at 80 ℃ for 1 week to allow compositional transformation from CaCO_3_ to CO_3_Ap granules (Gr)^10,31^.

### Preparation of bioresorbable mesh cages containing CO_3_Ap granules

**Figure 1:**
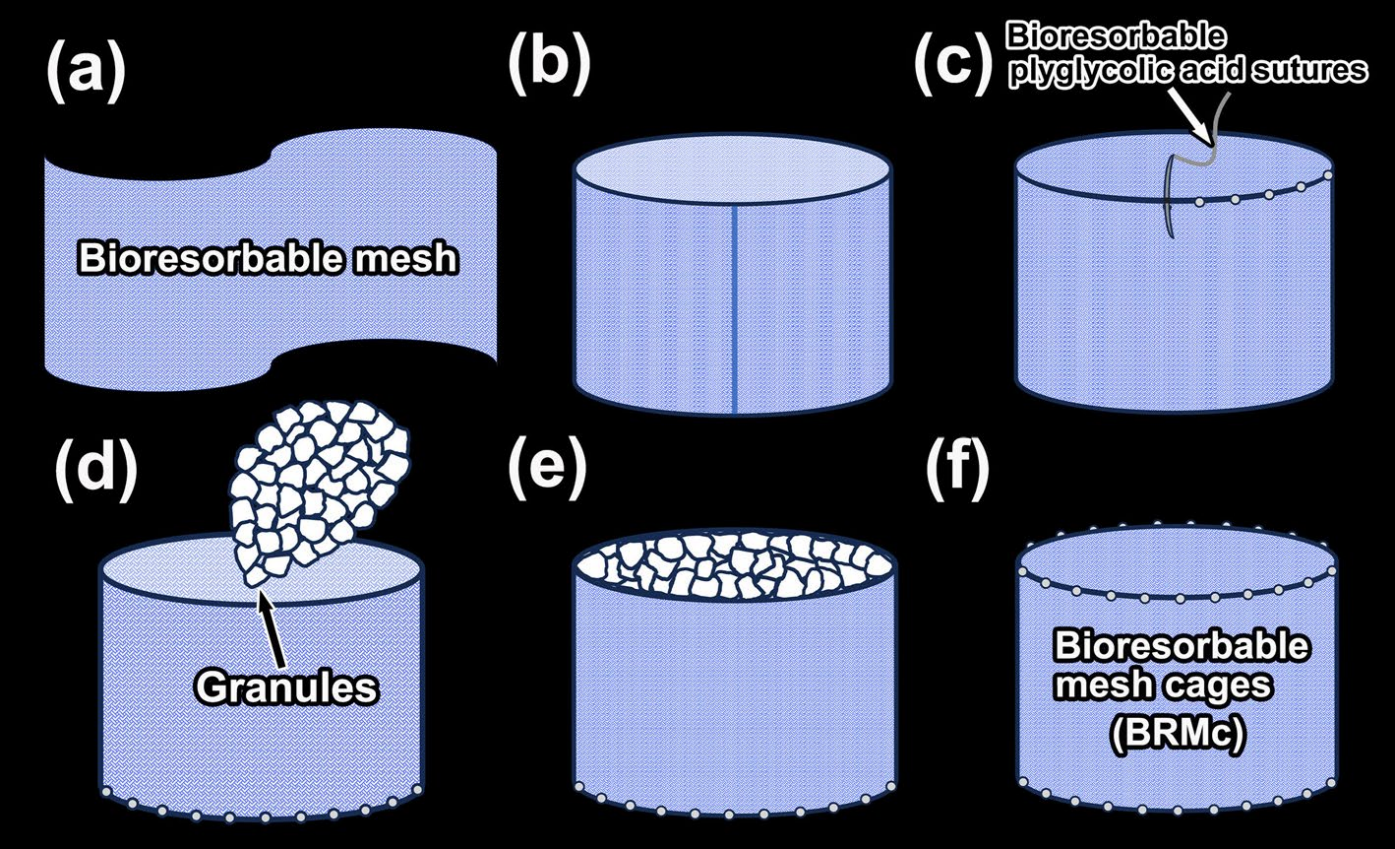
Preparing bioresorbable mesh cages (BRMc). Bioresorbable mesh cages (diameter, 6 mm; length, 4 mm) were cylindrical formations and used bioresorbabole mesh of Vicryl mesh knitted type and bioresorbable polyglycolic acid sutures. The hollow cylinder was formed from Vicryl mesh sheet (**a**) such that the edge of the sheet was joined by heat sealer (**b**). One side of the hollow cylinder was joined with circumference of Vicryl mesh sheet (diameter, 6 mm) at multiple spots by the bioresorbable sutures (**c**). CO_3_Ap granules were introduced from the other side against sutured sheet (**d**). Then the granules introduced side of hollow cylinder (**e**) was joined with circumference of Vicryl mesh sheet (diameter, 6 mm) at multiple spots by bioresorbable sutures (**f**).

Bioresorbable mesh cages (BRMc) (diameter, 6 mm; length, 4 mm) were cylindrical formations and used bioresorbabole mesh of Vicryl mesh knitted type (Ethicon, Somerville, NJ, USA)^32^ and bioresorbable polyglycolic acid sutures (diameter, 0.07–0.99 mm) (Alfresa Pharma Osaka, Japan). The hollow cylinder was formed from Vicryl mesh sheet (a) such that the edge of the sheet was joined by heat sealer (b). One side of the hollow cylinder was joined with circumference of Vicryl mesh sheet (diameter, 6 mm) at multiple spots by the bioresorbable sutures due to prevent the granules leak from the cages (c) and CO_3_Ap granules were introduced from the other side against sutured sheet (d). Then the granules introduced side of hollow cylinder (e) was joined with circumference of Vicryl mesh sheet (diameter, 6 mm) at multiple spots by bioresorbable sutures (f) (Fig. 1).

### Ethics statement

The animal study in this study were approved by the Animal Care and Use Committee of Kyushu University (approval number and date: A20-358-0, February 25, 2021). This study was done in compliance with the Animal Research: Reporting In Vivo Experiments (ARRIVE) guidelines. The study was conducted in accordance with relevant guidelines and regulations.

### Animals

Japanese white rabbits (Japan SLC, Inc., Shizuoka, Japan) aged 18 weeks and weighing 3.0–3.5 kg were used for the experiments. The rabbits were housed in the Center of Biomedical Research, Research Center for Human Disease Modeling, Graduate School of Medical Sciences, Kyushu University. In total, 10 rabbits were used (5 rabbits per week (4 weeks, 12 weeks)).

### Surgical procedure

The rabbits were subjected to general anesthesia by intraperitoneal injection of ketamine (30 mg/kg, Daiichi Sankyo Co., Ltd, Tokyo, Japan) and xylazine (5.0 mg/kg, Elanco Japan, Co., Ltd, Tokyo, Japan). After shaving the region of the rear limbs of the rabbits and disinfecting the skin with 10% povidone-iodine solution (Meiji Seika Pharma Co., Ltd., Tokyo, Japan), 2% lidocaine (Dentsply Sirona Co., Ltd., Tokyo) was administered as local anesthesia. The cavities of critical-sized cylindrical defects (6 mm diameter, 4 mm depth) were created in both femoral medial condyle of each rabbit by drilling with a trephine bur. After irrigation of the bone defects hole and graft sample of Gr or BRMc/Gr with sterile saline, Gr or BRMc/Gr were implanted into the cavity of each femur, respectively. After implantation, the periosteum and skin flap was repositioned and sutured using nylon sutures. The surgical site was again disinfected with 10% povidone-iodine (Meiji Seika Pharma Co., Ltd). Gentamicin sulfate (GENTACIN®, MSD Co., Japan) was injected to prevent infection. The femoral condyle was not immobilized after surgery, and the rabbits were allowed to move freely in their cages. At 4 and 12 weeks after the implantation of the Gr and BRMc/Gr, the rabbits were euthanized with an anesthetic overdose, and both femurs were collected (the total number of samples was 20 (5 samples per group (Gr (4 weeks), BRMc/Gr (4 weeks), Gr (12 weeks), and BRMc/Gr (12 weeks)).

### Micro-computed tomography (micro-CT)

The remaining materials in the bone defect and outside of the bone defect area were evaluated using microcomputed tomography (micro-CT; SkyScan; Bruker Corporation, MA, USA) at source voltage and current of 59 kV and 169 μA, respectively. The images were reconstructed using the NRecon software (Skyscan).

### Histomorphological analysis

The harvested femurs were fixed in 10 wt% buffer formaldehyde (Wako Pure Chemical) for 7 days. The sample were decalcified 0.5 mol/L etylenediaminetetraacetic acid, and embedded in paraffin for histological analyses and sectioned coronally. Sections were stained with hematoxylin and eosin (HE) (New bone was stained by HE and distinguished from other areas) and examined under a microscope (BZ-X710, Keyence, Osaka, Japan). The area% of the new bone formed in each defect was calculated using Eq. (1), and the area% host bone was calculated using Eq. (2). The host bone area was detected as the area being host bone positioned lateral condyle not created bone defect of the femur and the area range were as same as inside bone defect area created medial condyle of the femur,

1 $$ {\text{Area}}\% {\text{ of new bone formed in each defect }} = \, \left( {{\text{new bone formed area in the bone defect}}/{\text{bone defect area}}} \right) \, \times {1}00, $$

2 $$ {\text{Area}}\% {\text{ of host bone }} = \, \left( {{\text{host bone area }}/{\text{ bone defect area}}} \right) \, \times { 1}00. $$

New bone in defects were quantified two-dimensionally using ImageJ version 1.43 (National Institutes of Health, Bethesda, MD, USA) public domain software.

### Statistical analysis

At 4 and 12 weeks after implantation, the area% of remaining materials and newly formed bone was analyzed statistically using Excel v. X (Microsoft Co., Redmond, Washington, USA). The chi-squared test was used to investigate whether each group had a normal distribution. Bartlett’s test was employed to examine the homogeneity of variance across samples. The result from the chi-squared and Bartlett’s tests concerning the area% of remaining materials, Kruskal–Wallis tests were used to assess the difference among each group. The area percentages of the remaining materials were presented as boxplots of the median with 25% and 75% quartiles. Whisker indicates minimum and maximum. Statistical significance was considered as p-value < 0.05. In cases where significant differences were detected values, Steel–Dwass multiple comparison analysis would be used. The result from the chi-squared test and Bartlett’s tests concerning the area% of newly formed bone, a one-way analysis of variance was used to assess the difference among each group. Statistical significance was considered as p-value < 0.05. In cases where significant differences were detected in the mean values, Tukey–Kramer multiple comparison analysis would be used as a post hoc test. All values of the area percentages of the newly formed bone were reported as mean ± SD (standard deviation).

## Results

### Characterization of CO_3_Ap introduced into bioresorbable mesh cages

**Figure 2:**
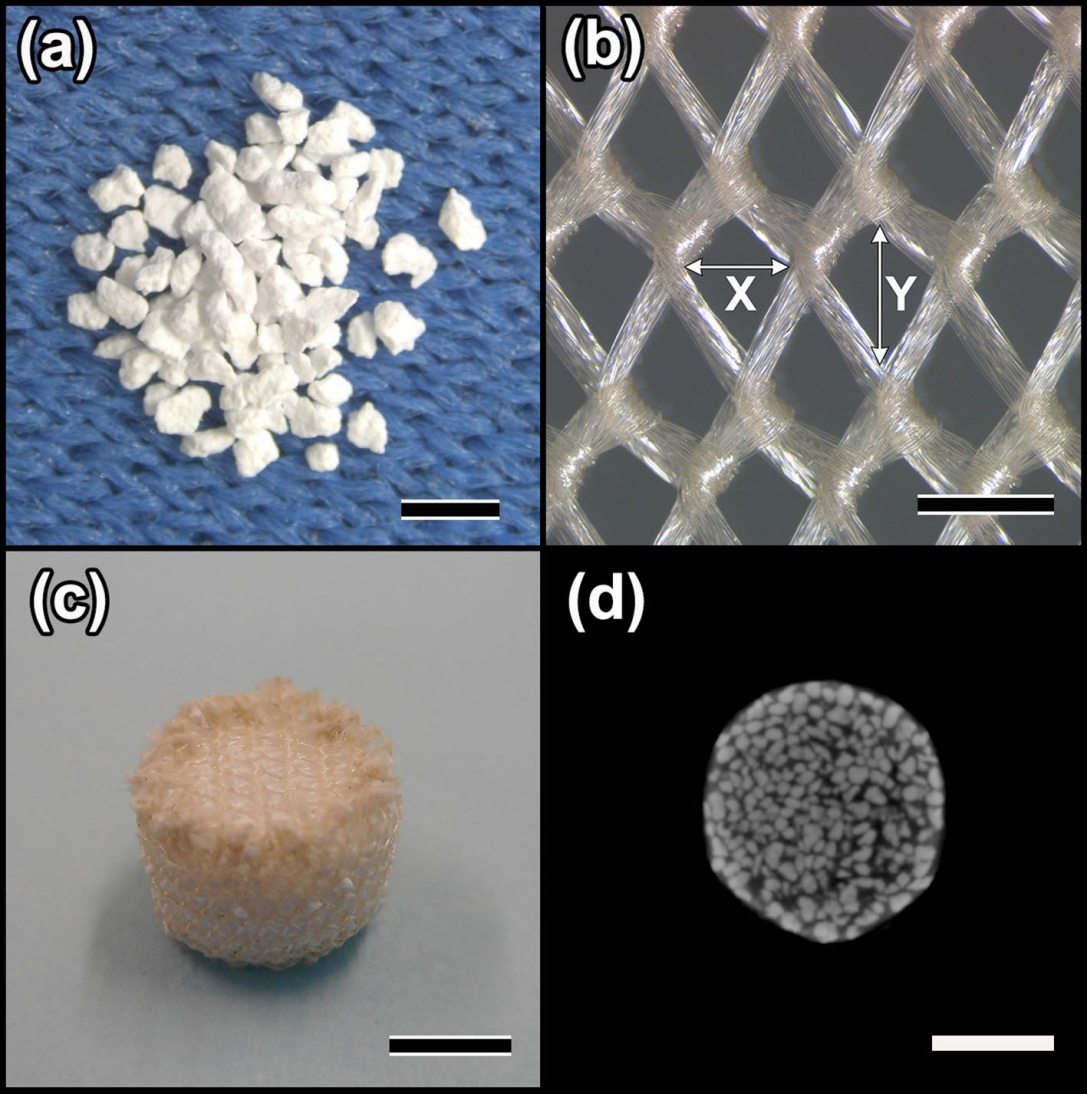
Characterization of CO_3_Ap introduced into bioresorbable mesh cages (BRMc). Bioresorbable mesh cages containing bone graft granules (BRMc/Gr) were made from CO_3_AP granules (**a**), Vicryl mesh knitted type (**b**), and bioresorbable polyglycolic acid sutures. The pore of Vicryl mesh was rhomb and the maximum horizontal, diagonal length was 333.0 ± 10.5 μm ((**b**) X) and the maximum vertical, diagonal length was 502.0 ± 20.7 μm ((**b**) Y). BRMc were cylindrical formation (6 mm diameter, 4 mm length) and CO_3_Ap granules were introduced into BRMc during their preparation (BRMc/Gr (**c**)). Micro-CT images of BRMc/Gr provided cross-sectional views of the images (**d**). The scale bars of (**a–d**) indicate 1 mm, 500 μm, 3 mm, and 3 mm, respectively.

BRMc were made from CO_3_Ap granules CO_3_Ap granules (Fig. 2a), Vicryl meshes knitted type (Fig. 2b), and bioresorbable polyglycolic acid sutures. The pore of Vicryl mesh was rhomb and the maximum horizontal, diagonal length (Fig. 2bX) was 333.0 ± 10.5 μm, and the maximum vertical, diagonal length (Fig. 2bY) was 502.0 ± 20.7 μm. BRMc were cylindrical in formation (6 mm diameter, 4 mm length) and made from Vicryl mesh and bioresorbable sutures. CO_3_Ap granules were introduced into BRMc during their preparation (BRMc/Gr) (Fig. 2c). Micro-CT images of BRMc/Gr provided cross-sectional views of the images (Fig. 2d). The pore ratio of the CO_3_Ap granules was evaluated using the volume and weight of each sample and theoretical density. Because the theoretical density of CO_3_Ap was unknown, that of HAp (3.16 g/cm^3^) was used to calculate CO_3_Ap porosity. The average porosities in BRMc/Gr were 65%, which were similar to those of Gr (67%) inserted into the stainless-steel vessel (6 mm diameter, 4 mm length) and imitated bone defect. No deviations of the granules from the mesh cages were observed. The bone defects (6 mm diameter, 4 mm length) were created by a trephine bur in the femoral condyles of the rabbits and implanted Gr or BRMc/Gr respectively in each the femoral condyles of the rabbits.

### Micro-CT analysis

**Figure 3:**
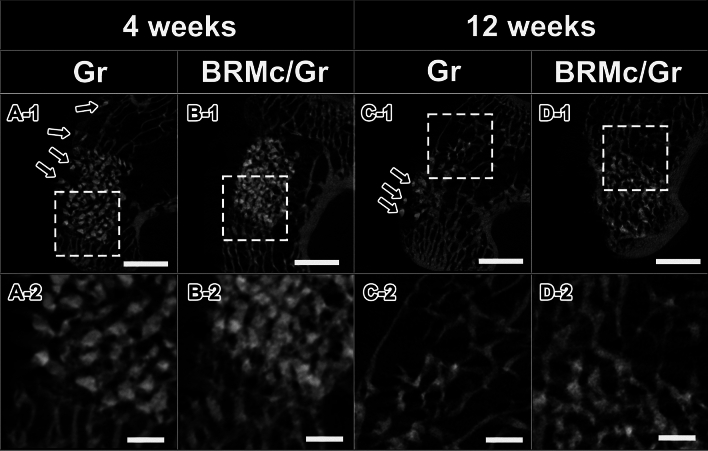
Micro-CT images at 4 and 12 weeks after implantation. Micro-CT images of rabbit femurs with defects were corrected by grafting using Gr and BRMc/Gr at 4 weeks (**A-1,B-1**) and 12 weeks (**C-1,D-1**). (**A-2,B-2,C-2,D-2**) are magnified images of the dotted line of (**A-1,B-1,C-1,D-1**). The scale bars of (**A-1,B-1,C-1,D-1**) indicate 3 mm, respectively. The scale bars of (**A-2,B-2,C-2,D-2**) indicate 500 μm, respectively. The black arrowheads indicate the granules scattered outside of the bone defect (**A-1,C-1**).

**Figure 4:**
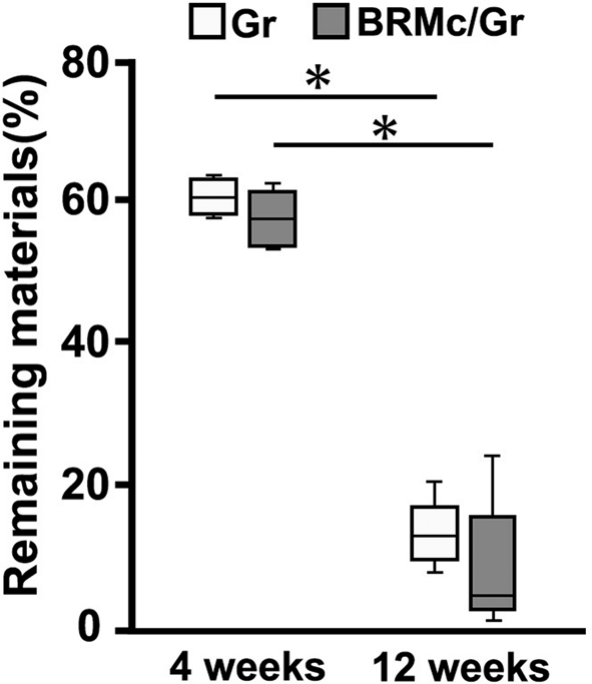
The area percentages of the remaining materials. Boxplot (Gr (white squares) and BRMc/Gr (gray squares)) show the remaining materials for both groups. The boxplots illustrate the median with 25% and 75% quartiles, respectively. Whisker indicates minimum and maximum. At 4 and 12 weeks, there was no significant difference between the remaining materials of Gr and BRMc/Gr respectively. At 12 weeks in Gr and BRMc/Gr, the area percentages of the remaining materials were significantly lower than at 4 weeks respectively. Statistical significance was considered as p-value < 0.05.

Four weeks after implantation in the Gr and BRMc/Gr, there was a large amount of radiopaque granules in the bone defect. Radiopaque granules were clearly distinguished from the lamellar (host bone) radiopaque figures around the bone defects (Fig. 3(A-1),(B-1)). Radiopaque granules were connected to elongated islet (host bone) radiopaque figures around the bone defect (Fig. 3(A-2),(B-2)). In Gr, some granular radiopaque figures were scattered outside the bone defect (Fig. 3(A-1)). In the BRMc/Gr, no granular radiopaque figures were observed outside the bone defect (Fig. 3(B-1)). At 12 weeks after implantation in Gr and BRMc/Gr, the amount of granular radiopaque figures in bone defects decreased compared with that at 4 weeks. These radiopaque granule figures were dotted in the lamellar radiopaque figures (Fig. 3(C-1),(D-1)). The regions of the radiopaque granules area in the bone defects and lamellar (host bone) radiopaque figures were obscure (Fig. 3(C-2),(D-2)). In Gr, some granular radiopaque figures were scattered outside the bone defect (Fig. 3(C-1)). In the BRMc/Gr, some granular radiopaque figures were observed skin side of the bone defect. However, it was considered that no granular radiopaque figures were outside of the bone defects because radiopaque figures were not clear outside of the bone defects compared with Gr, and the size of granular radiopaque was smaller than that of Gr as granules were absorbed (Fig. 3(D-1)). In Gr, the number of granular radiopaque figures scattered outside the bone defect was confirmed as 5 samples among 5 samples; in BRMc/Gr was confirmed as 0 samples among 5 samples. At 4 and 12 weeks after implantation about the area percentages of the remaining materials, there was no significant difference between Gr and BRMc/Gr respectively. At 12 weeks after implantation, the area percentages of the remaining materials of Gr and BRMc/Gr were significantly lower than their remaining materials at 4 weeks respectively (Fig. 4).

### Histological results of implants

**Figure 5:**
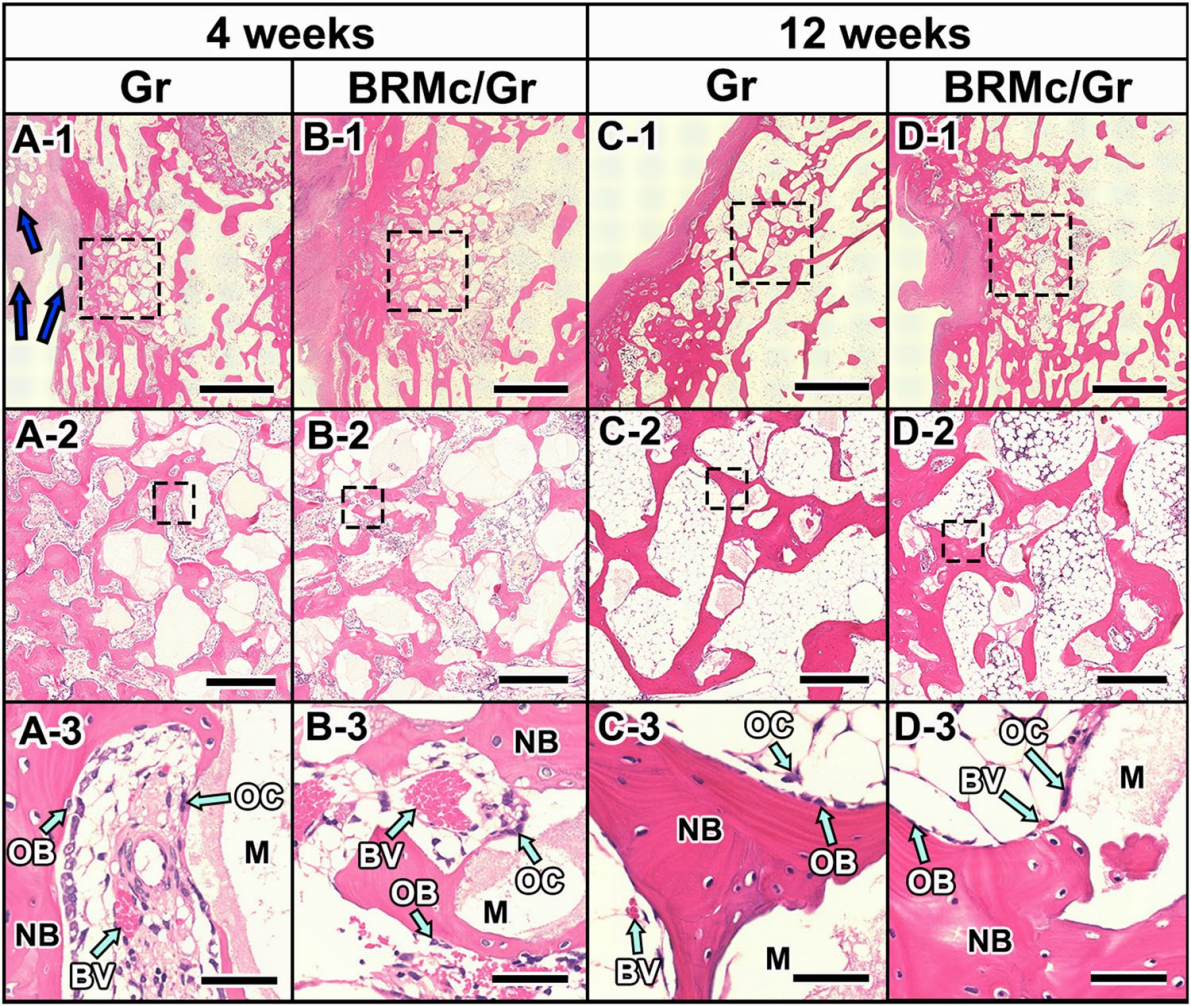
Histological examination of implants. Hematoxylin and eosin (HE) staining histological images of rabbit femurs with bone defects corrected by grafting with Gr, BRMc/Gr at 4 and 12 weeks after implantation. (**A-3,B-3,C-3,D-3**) are magnified images of the dotted line of (**A-2,B-2,C-2,D-2**), respectively. (**A-2,B-2,C-2,D-2**) are magnified images of the dotted line of (**A-1,B-1,C-1,D-1**), respectively. “NB”, “BV”, “OB”, “OC”, and “M” indicate newly formed bone, blood vessel, osteoblast, osteoclast, and materials, respectively. The scale bars of (**A-1,B-1,C-1,D-1**) indicate 2 mm, respectively. The scale bars of (**A-2,B-2,C-2,D-2**) indicate 500 μm, respectively. The scale bars of (**A-3,B-3,C-3,D-3**) indicate 100 μm, respectively. The blue arrowheads indicate the granules scattered outside of the bone defect (**A-1**). The green arrowheads indicate the BV, OB, and OC (**A-3,B-3,C-3,D-3**).

Four weeks after implantation in the Gr and BRMc/Gr, there was a large amount of granules and newly formed bone in the bone defect. In Gr and BRMc/Gr, new bone was formed from the marginal area to the central area of the bone defects (Fig. 5(A-1),(B-1)). In BRMc/Gr, the bioresorbable mesh or suture were not completely resorbed (Fig. 5(B-1)). The newly formed bone was observed around the granules (Fig. 5(A-2),(B-2)). In Gr, some granules were scattered outside of the bone defect (Fig. 5(A-1)). In Gr and BRMc/Gr, there were fibrous tissue layers at the skin side and minimal signs of local tissue inflammation. There were biodegraded mesh or sutures around granules in BRMc/Gr. The blood vessels (BV) were near the newly formed bone (NB), and osteoblasts (OB) were present on the surfaces of the newly formed bone (NB), while osteoclasts (OC) were near the material (M) (Fig. 5(A-3),(B-3)). At 12 weeks after implantation in Gr and BRMc/Gr, the amount of granules inside the bone defects decreased compared with that at 4 weeks. New bone was formed around the granules, and the form of the new bone was similar to that of the host bone outside the bone defect. The bioresorbable mesh was resorbed (Fig. 5(C-1),(D-1)). The granules rounded by newly formed bone were absorbed compared with that at 4 weeks (Fig. 5(C-2),(D-2)). There were minimal signs of local tissue inflammatory foreign body reaction compared with 4 weeks in Gr and BRMc/Gr. In BRMc/Gr, there was no mesh or suture remaining. The blood vessels (BV) were near the newly formed bone (NB). Osteoblasts (OB) were present on the surfaces of the newly formed bone (NB), while osteoclasts (OC) were near the material (M). The number of these cells were lower compared to that at 4 weeks (Fig. 5(C-3),(D-3)).

### Histomorphometrical examination

**Figure 6:**
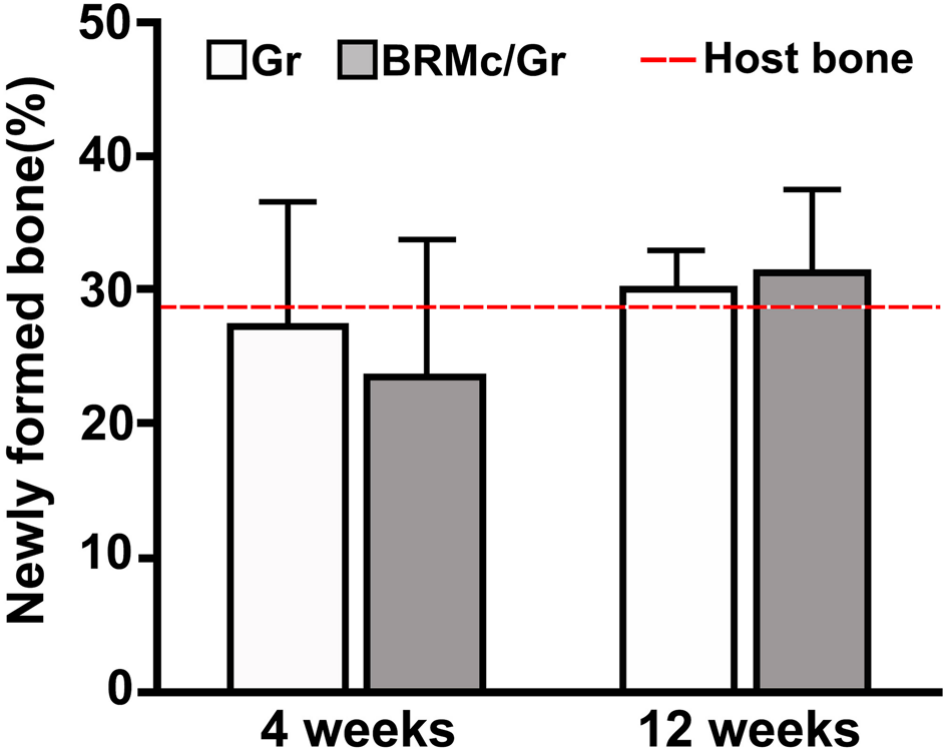
Histomorphometric examination of the percentage of newly formed bone. The means of percentage volume of the newly formed bone (newly formed bone%) ± standard deviation is shown in bar graphs (Gr (white squares) and BRMc/Gr (gray squares)). At 4 and 12 weeks, there was no significant difference between the newly formed bone of Gr and BRMc/Gr. At 12 weeks in Gr and BRMc/Gr, the newly formed bone was higher than at 4 weeks, but there was no significant difference between 4 and 12 weeks. The percentages of host bone were 28.2% ± 7.1% and the dotted line refers to the means of the host bone (28.2%).

At 4 weeks, the percentages of newly formed bone intrabony defects in Gr and BRMc/Gr were 26.9% ± 9.6% and 23.4% ± 10.3%, respectively. There was no significant difference between Gr and BRMc/Gr. At 12 weeks, the percentages of newly formed bone intrabony defects in Gr and BRMc/Gr were 30.1% ± 3.0% and 31.3% ± 6.5%, respectively. There was no significant difference between Gr and BRMc/Gr. At 12 weeks in Gr and BRMc/Gr, the newly formed bone was higher than 4 weeks, but, there was no significant difference between 4 and 12 weeks. The percentages of host bone were 28.2% ± 7.1%. There was no significant difference with Gr and BRMc/Gr (Fig. 6).

## Discussion

This study indicated that osteoprogenitors in the bloodstream from host bone or periosteum passed through pore of BRMc and penetrated the porous structure of graft materials (granules) and the osteoprogenitors might have interacted with individual granules of graft materials. Then bone remodeling could progress actively and new bone was formed. The newly formed bone was similar to the host bone at 12 weeks after implantation. BRMc/Gr could reduce the risk of unwanted new bone formation occurring due to leakage and loss of granules from the bone defects compared with Gr because BRMc enclosed granules and prevent granules leakage from bone defects after implantation. Additionally, BRMc/Gr could keep granules assembled in one place and improved carried easily.

At 4 weeks after implantation, large amounts of granules remained of the bone defect in Gr and BRMc/Gr (Fig. 4). New bone of Gr (26.9% ± 9.6%) and BRMc/Gr (23.4% ± 10.3%) was formed from the marginal area to the central area of the implanted samples, and newly formed bone was formed around the granules. A large number of blood vessels (BV) that efficiently delivered nutrients and oxygen were found near the new bone. Moreover, a large number of osteoblasts (OB) and osteoclasts (OC) inducing bone remodeling were present near the granules and new bone, respectively. At 12 weeks after implantation, small amounts of granules remained in the bone defect in Gr and BRMc/Gr (Fig. 3). Newly formed bone of Gr (30.1% ± 3.0%) and BRMc/Gr (31.3% ± 6.5%) was mature lamellar bone similarly to host bone (28.2% ± 7.1%). The boundary between new bone inside of bone defect and the host bone became unclear. There were signs of minimal inflammation in Gr and BRMc/Gr. It has previously been reported that few inflammatory cells and minimal fibrotic reaction around the graft were observed at 12 weeks after transplantation when islets in thrombin–fibrin gel sandwiched between layers of Vicryl mesh were transplanted into the subcutaneous site in rats. High endothelial cell density around the graft was maintained^33^. Additionally, there was no evidence of residual chronic inflammatory process at 12 weeks after insertion when hydroxyapatite-coated metal implant the interface of the whole length of which augmented with a slurry of autogenous cancellous bone chips and marrow packed using Vicryl mesh was inserted into bone defects and the interface was attached tendons. The tendon-bone interface regions had reorganized^22^. Thus, it was considered that in the case of long-term grafts, such as 12 weeks, we considered inflammatory reaction was alleviated and became minimal. Therefore Vicryl mesh could not induce unfavorable effects on graft. A small number of blood vessels (BV) were found near the new bone, and a small number of osteoblasts (OB) and osteoclasts (OC) were present near the materials and new bone, respectively.

Thus, at 4 weeks after implantation, a large number of osteoblasts (OB) were present on the surfaces of the newly formed bone (Fig. 5(A-3),(B-3)). There was no significant difference in new bone volume between Gr and BRMc/Gr (Fig. 6). Additionally, from 4 to 12 weeks after implantation, the materials were absorbed and replaced newly formed bone, the formation of new bone was similar to the host bone, and the volume of newly formed bone was increased. As suggested in a previous study, many microvessels penetrating through the Vicryl mesh structure were observed, and blood cells and endothelial cells were visualized in the surrounding tissue of the islet graft using the mesh^33^. Additionally Carbonate apatite (CO_3_Ap), which is the main component of the bone mineral, resorbed by the osteoclasts at a similar pace as that of new bone formation and can thus be replaced with new bone. In addition, CO_3_Ap was found to upregulate differentiation of osteoblast and the osteoconductivity of CO_3_Ap is much higher than that of HA and β-TCP^30,31,34^. These indicated that after implantation, osteoprogenitors in the bloodstream from the host bone or in the periosteum had passed through the pore of BRMc, penetrating the porous structure of graft materials (granules of CO_3_Ap) enclosed by BRMc and progressed bone remodeling actively. Newly formed bone increased thanks to the interaction between individual granules of graft materials and osteoprogenitor cells from 4 to 12 weeks and became matured bone, like host bone.

From the micro-CT analysis at 4 and 12 weeks, the leakage of granules from bone defects was observed in Gr (5 samples among 5 samples), whereas no granules were observed around the bone defects in BRMc/Gr (0 samples among 5 samples). This demonstrates that BRMc could prevent granule leakage outside the mesh cages and from bone defects. Althogh there was no significant difference in remaining materials between Gr and BRMc/Gr in this study, the remaining materials of Gr tended to be larger than BRMc/Gr. It could be considered that Gr included granules leaked outside from bone defects and the resorption of the granules could be lower and remained compared with that of granules inside bone defects. In addition, there was no significant difference in newly formed bone between Gr and BRMc/Gr after 12 weeks implantation; however newly formed bone of BRMc/Gr tended to be larger than Gr. It has been previously reported that the early loss of biomaterial granules during wound healing hampers the regeneration^35^. Another report suggested that the bone volume that covered the surface of the bone defects when using Vicryl mesh was greater than the bone volume that did not cover the surface when granules were implanted into bone defects. It was indicated that Vicryl mesh had prevented leak granules that could progress bone remodeling from the bone defects because the former was retained inside the bone defects compared with the latter was leaked and loss of graft materials^11^. Additionally, it could be considered that the amount of newly formed bone in Gr were less increased compared with BRMc/Gr from 4 to 12 weeks after implantation because Gr included granules not progressed bone remodeling leaked outside from bone defects in this study. These indicate that BRMc/Gr could reduce the risk of unwanted new bone formation occurring by leak and loss of granules from bone defects compared with Gr because BRMc could enclose granules and prevent granules leakage from bone defects after implantation. Additionally BRMc could not induce unfavorable effects to forme new bone because minimal signs of local tissue inflammatory foreign body reaction. The other advantage of BRMc/Gr is that they could keep granules gathered in one place, avoid displacement of granules to unintended locations, and became carry easily compared with Gr. Therefore, BRMc/Gr was effective in bone regeneration and improved clinical handling.

## Conclusion

In this study, we fabricated a bioresorbable mesh cages containing bone graft granules composed of carbonate apatite (BRMc/Gr). The samples were implanted into bone defect in the rabbit femur. Micro-computed tomography and histological analysis was conducted at 4 and 12 weeks. Osteoprogenitor cells passed through the pores of BRMc from outside into inside of the pore of BRMc and penetrated the porous structure of graft materials (granules) enclosed by BRMc, and the osteoprogenitors might have interacted with individual granules of graft materials. Then bone remodeling could progress actively, and new bone was formed. The formation of new bone of BRMc/Gr became similar to the host bone and there were minimal signs of local tissue inflammation at 12 weeks after implantation. BRMc/Gr could reduce the risk of unwanted new bone formation occurring due to leakage and loss of granules from the bone defects compared with Gr because BRMc could enclose granules and prevent granules leakage from the bone defects and BRMc could not induce unfavorable effects to forme new bone after implantation. Additionally, BRMc/Gr could keep granules assembled in one place, avoid displacement of granules to unintended locations, and carry easily. Therefore, BRMc/Gr was effective in bone regeneration and improved clinical handling.

## Data Availability

All data generated or analysed during this study are included in this published article.
